# Multi-speckle X-ray photon correlation spectroscopy in the ultra-small-angle X-ray scattering range

**DOI:** 10.1107/S1600577516008092

**Published:** 2016-06-15

**Authors:** Johannes Möller, Yuriy Chushkin, Sylvain Prevost, Theyencheri Narayanan

**Affiliations:** a ESRF – The European Synchrotron, 38043 Grenoble, France

**Keywords:** USAXS, XPCS

## Abstract

Multi-speckle X-ray photon correlation spectroscopy (XPCS) measurements in the ultra-small-angle range are performed using a long pinhole collimation instrument in combination with two-dimensional photon-counting and high-sensitivity imaging detectors. The feasibility of the presented setup to measure dynamics on different time and length scales pertinent to colloidal systems is shown. This setup offers new research opportunities, such as for example in the investigation of non-equilibrium dynamics in optically opaque, complex systems over length scales from tens of nanometres to several micrometres. In addition, due to the short duration of the X-ray exposure involved in the ultra-small-angle range, possible radiation-induced effects are alleviated. Furthermore, the performance of two different detectors, a photon-counting Pilatus 300K and an integrating FReLoN CCD, are compared, and their applicability for accurate XPCS measurements is demonstrated.

## Abstract 2

The feasibility for multi-speckle XPCS measurements in the ultra-low-angle range is demonstrated. The performance of the setup is illustrated by means of colloidal dynamics.

## 1. Introduction   

For nearly half a century, complex dynamics and relaxations in various condensed matter systems have been studied by using coherent visible-light scattering techniques, most prominently dynamic light scattering (DLS) (Berne & Pecora, 2000 ▸). In this method, the dynamics within the material are manifested by the fluctuations in the coherent scattering intensity. The same principle also holds when the dynamics are probed by using a coherent X-ray beam. This technique is known as X-ray photon correlation spectroscopy (XPCS) (Mainville *et al.*, 1997 ▸; Grübel *et al.*, 2008 ▸; Sutton, 2008 ▸). With the increasing brilliance of synchrotron radiation sources over the last decades, numerous applications of coherent X-ray scattering methods have emerged. Among them, XPCS has been well established as a technique to probe slow dynamics in complex systems (Grübel *et al.*, 2008 ▸; Sutton, 2008 ▸; Madsen *et al.*, 2010 ▸).

One main assumption in the treatment of DLS and XPCS data is that every detected photon has only been scattered once by the sample, *i.e.* there is no multiple scattering. In the case of light scattering however, especially when used for studying concentrated systems with sizes up to several micrometres or with high refractive index contrasts, multiple scattering becomes highly probable (Segre *et al.*, 1995*a* ▸; Moussaïd & Pusey, 1999 ▸). This needs further technical development in order to either suppress the multiple scattering as in cross-correlation methods (Segre *et al.*, 1995*b* ▸; Aberle *et al.*, 1998 ▸; Block & Scheffold, 2010 ▸) or to restrict the analysis to the strongly multiple-scattering regime by diffusing wave spectroscopy (Pine *et al.*, 1988 ▸). The conventional DLS is therefore often limited to low concentrations and not readily suitable for samples which are optically opaque or highly absorbing. This limitation can be overcome by the use of hard X-rays, which have a much smaller scattering probability, making XPCS a valuable technique for optically opaque systems.

However, the information in the scattered intensity on a certain length scale relates to the scattering at a certain angle, given by the scattering vector of magnitude *q*, which has units of reciprocal length. Here, with θ being the scattering angle and λ the wavelength of the incoming photons,
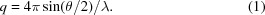
Therefore, scattering from the same length scale occurs at much smaller angles when the photon energy is increased from visible light to X-rays. In order to access length scales in the micrometre range, scattering vectors down to 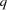 = 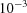
 nm^−1^ are required. This is usually difficult to reach experimentally for typical X-ray wavelengths (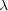 ≃ 1 Å).

A standard experimental setup for XPCS, or coherent scattering in general, at a modern synchrotron beamline consists of a small-angle X-ray scattering (SAXS) pinhole camera in combination with a high-resolution two-dimensional detector (Abernathy *et al.*, 1998 ▸; Sandy *et al.*, 1999 ▸; Llopart *et al.*, 2002 ▸; Westermeier *et al.*, 2009 ▸; Shinohara *et al.*, 2010*a* ▸; Hoshino *et al.*, 2012 ▸). Since the synchrotron is only a partially coherent source, a high degree of spatial filtering is needed to obtain a transverse coherence length of several tens of micrometres (Abernathy *et al.*, 1998 ▸; Sandy *et al.*, 1999 ▸; Alaimo *et al.*, 2009 ▸). Different constraints determine the smallest accessible *q* value in such a setup, like the maximum sample-to-detector distance, natural divergence of the X-ray beam, pixel sizes and intensity dynamic range of the available detectors, the limited numbers of pixels near to the direct beam and the size of the beamstop itself, which has to be used in order to protect the detector from the direct beam. Of course, these limitations hold true for dynamic as well as static small-angle scattering experiments. Therefore, the smallest reported *q* values for pinhole geometry SAXS setups are of the order of 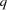 = 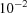
 nm^−1^.

This issue has been addressed in the past with the construction of dedicated Bonse–Hart setups, which have successfully been employed for ultra-small-angle X-ray scattering (USAXS) experiments (Sztucki *et al.*, 2006 ▸; Sztucki & Narayanan, 2007 ▸; Zhang *et al.*, 2011 ▸). A Bonse–Hart double-crystal setup at an undulator source offers the high angular resolution needed for reaching the USAXS range as well as the coherent flux to perform XPCS experiments in this geometry (Sztucki *et al.*, 2006 ▸; Zhang *et al.*, 2011 ▸, 2012 ▸). However, this kind of setup has certain drawbacks since it involves a sequential acquisition at each scattering vector by a point detector. This can be a problem when investigating anisotropic and radiation-sensitive or non-equilibrium samples, which is often the case for typical soft matter systems. In contrast, with a two-dimensional detector, data over a wide range of scattering vectors and the full azimuthal range are simultaneously acquired. In addition, a direction-dependent analysis (Fluerasu *et al.*, 2010 ▸; Burghardt *et al.*, 2012 ▸) can be made more easily by using a two-dimensional detector. It is worth mentioning that static scattering information as well as *q*-dependent relaxation times for even smaller scattering vectors (*q* < 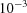
 nm^−1^) can, in principle, be obtained by analyzing the near-field speckle patterns (Cerbino *et al.*, 2008 ▸). This analysis is, however, often complicated due to the complex spatial response function of the detector (Alaimo *et al.*, 2009 ▸; Lu *et al.*, 2011 ▸).

In this paper, we demonstrate XPCS measurements in the USAXS range using a pinhole geometry, which became available at the recently upgraded beamline ID02 at the ESRF (Van Vaerenbergh *et al.*, 2015 ▸). The new focusing scheme and the new 34 m-long flight tube equipped with different two-dimensional detectors allow access to scattering angles corresponding to length scales of several micrometres. This setup offers not only the unique opportunity to perform time-resolved USAXS but also USAXS-XPCS experiments using two-dimensional detectors, which is the subject of this paper.

## 2. X-ray photon correlation spectroscopy   

### 2.1. Coherence and contrast   

XPCS probes the dynamic properties of matter through the temporal correlation of the scattering intensity. The homodyne intensity–intensity time autocorrelation function is defined as
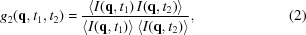
with 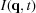 being the intensity measured at wavevector 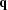 at time *t* and 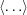 the pixel average (Berne & Pecora, 2000 ▸). With this so-called two-time correlation function, non-equilibrium and non-stationary dynamics can be investigated (Brown *et al.*, 1997 ▸; Fluerasu *et al.*, 2007 ▸; Shinohara *et al.*, 2010*b* ▸). For limited time intervals, however, when the overall measurement time is much smaller than typical aging times in the sample or in the case of a stationary system (*e.g.* Brownian motion), the two-time correlation function can be described for a (quasi-)stationary state as (Berne & Pecora, 2000 ▸)
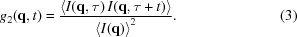
Here, 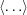 denotes the time averaging. In the limit of large times 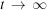, the value of 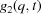 approaches unity, as the scattering signals over large time scales are completely uncorrelated. For short delay times 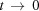, however, it tends to 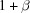, with β being the so-called contrast, which is strongly connected to the coherence properties of the radiation, and 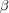 ≃ 1 for a single coherence volume.

With coherent illumination, the observed scattering pattern displays a typical speckle pattern, obeying negative exponential intensity statistics. The resulting probability distribution of the scattered intensity can then be written as (Goodman, 1985 ▸)
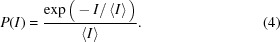
The opposite would be the case when the scattering volume is much larger compared with the coherence volume, so that 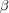 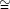 0. In this case, the intensity distribution follows Gaussian statistics.

At modern synchrotron radiation beamlines, a partially coherent beam of X-rays with a high flux is produced, which can be spatially filtered for coherent scattering experiments by using an aperture smaller than the coherence area of the beam (Jakeman *et al.*, 1976 ▸; Abernathy *et al.*, 1998 ▸; Sandy *et al.*, 1999 ▸). However, further experimental constraints alter the contrast of the XPCS experiment. In a typical experiment, with highly coherent radiation, the beamline optics, sample scattering geometry and the X-ray detector will influence the contrast. The optimum signal-to-noise ratio of 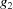 is obtained when the angular source size is the same as the angular acceptance of the detector pixels (Falus *et al.*, 2006 ▸). In the present case, the angular source acceptance is collimated to the similar size in both vertical and horizontal directions to obtain the highest *q* resolution. For partial coherence, the intensity probability distribution function 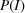 can be written as
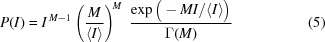
(Goodman, 1985 ▸), where *M* is the number of coherence volumes, which is related to the contrast as 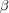 = 1/*M*.

### 2.2. XPCS data   

To demonstrate the XPCS capabilities of the presented setup, we study the free, Brownian diffusion of dilute colloidal particles. The mean-square displacement of the particles 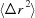 is related to the free diffusion constant 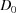,
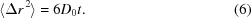
In this case, the intensity autocorrelation function can be written as a single exponential,
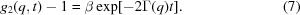
Here, 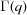 is the relaxation rate, or 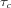 = 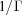 the relaxation time. The factor of 2 arises from the Siegert relation which relates the intensity autocorrelation function to the electric field correlation function (Berne & Pecora, 2000 ▸). For freely diffusing particles, the relaxation rate Γ is proportional to the diffusion coefficient,
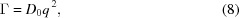
which is given by the Stokes–Einstein relation
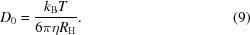
where *T*, η, 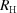 and 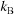 are the temperature, the viscosity of the suspending medium, the hydrodynamic radius of the particles and the Boltzman constant, respectively.

## 3. Experimental   

### 3.1. Materials   

Two different samples were used to demonstrate the XPCS capabilities of the presented setup; charge-stabilized silica particles with nominal diameters of 450 and 600 nm, produced *via* the Stöber synthesis. To increase the concentration of particles, the samples were left to sediment and then the lower part of the solution was used. The samples exhibited strong multiple light scattering, as they appeared milky, demonstrating the difficulty to investigate these samples with conventional DLS. The volume fraction of the 450 nm-sized particle suspension was estimated from the absolute scattering intensity to be 0.01.

In order to slow down the dynamics and test the presented setup on different timescales, one sample (600 nm) was transferred into a water/glycerol solution. The water to glycerol ratio was 76.5 wt%. Therefore, the viscosity of the medium is higher by roughly a factor of 41 as compared with pure water, slowing the diffusional motion down correspondingly. The volume fraction was obtained, taking into account the reduced contrast, as 0.004.

### 3.2. Beamline ID02, ESRF   

#### 3.2.1. Source and optics   

**Figure 1:**
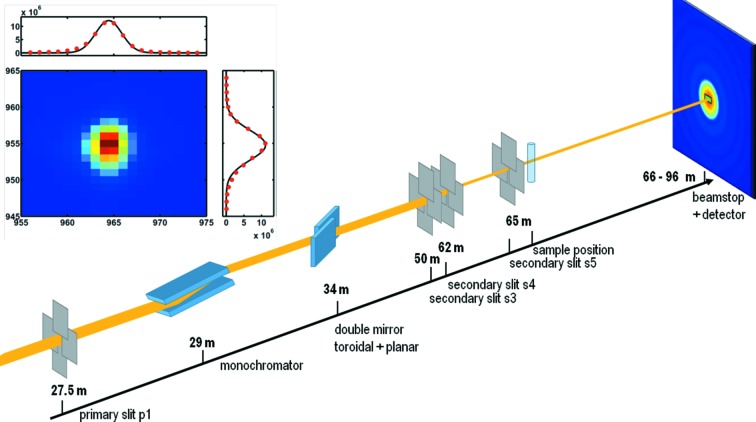
Sketch of the collimation scheme and experimental setup. The inset shows the attenuated direct beam at a distance of 30.7 m from the sample position, measured with the FReLoN detector.

The X-ray beam is delivered by two phased undulators of period 21.4 mm, length 1.6 m and minimum gap of about 11 mm. The central cone of the undulator spectrum is selected by primary slits of size 0.2 mm × 0.2 mm. The incident beam is monochromated (*E* = 12.46 keV) by a liquid-nitrogen-cooled channel-cut Si-111 monochromator (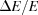 ≃ 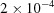). To preserve the source brilliance as much as possible, the focusing optics is based on a high-quality toroidal mirror reflecting in the horizontal plane. This makes the focused beam size less sensitive to longitudinal slope errors and a second horizontally reflecting fine planar mirror maintains the reflected beam parallel to the incident beam (Van Vaerenbergh *et al.*, 2015 ▸). Fig. 1 ▸ shows the positions of the main optical components. The beam is focused at the detector position about 96 m from the source.

#### 3.2.2. Detectors   

Two different detectors, a Pilatus 300K and a FReLoN (Fast-Readout, Low-Noise), were used to record the scattering data. The detectors differ in pixel size, readout time and detection principle. The Pilatus 300K is a single-photon-counting hybrid pixel detector and is therefore free of dark current and readout noise. It has an active area of 487 × 619 pixels, a pixel size of 172 µm × 172 µm and maximum repetition rate of 400 frames per second. The standard detector for USAXS experiments at ID02 is an integrating FReLoN CCD-based detector, which can be additionally used for XPCS experiments. It features a 20 µm-thick phosphor screen optically coupled to a Kodak KAF-4320 image sensor (2048 × 2048 pixels) *via* a fibre-optic plate. It offers smaller effective pixels sizes (24 µm) and therefore higher spatial resolution (∼48 µm) as well as single-photon sensitivity. However, the highest possible sampling rate, in 1 × 1 binning, is limited to about 5 Hz.

#### 3.2.3. Experimental setup   

XPCS measurements were performed with an X-ray energy of 12.46 keV. A sketch of the experimental setup is shown in Fig. 1 ▸. The inset shows the direct beam on the FReLoN detector.

The beam was collimated by a primary slit (p1) in front of the monochromator and two sets of secondary slits (s3, s4), which were set to aperture sizes of 40 µm × 50 µm and horizontally shifted by 35 µm, resulting in an effective slit aperture of 40 µm × 15 µm. Furthermore, a guard slit (s5), located 3 m from the last beam-defining slit, was used to clean parasitic scattering at ultra-low angles. With this arrangement, a well defined and collimated beam could be obtained. The size of the beam on the sample was 40 µm × 40 µm. At the detector position it was measured to be 64 µm × 99 µm (see inset of Fig. 1 ▸; the FWHM was measured to be 80 µm vertical and 110 µm horizontal, with the detector resolution of 48 µm). The resulting flux was 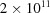
 photons s^−1^ and the sample-to-detector distance was 30.7 m. Different exchangeable beamstops of various size and shape are installed in the setup. But for XPCS, a circular beamstop of diameter either 1.6 mm or 3 mm was used which retained symmetrical azimuthal bins.

The colloidal samples were filled in quartz capillaries of 1 mm diameter and the temperature was controlled with a Peltier stage. The temperature was kept constant at 20°C for the duration of the whole experiment.

### 3.3. USAXS-XPCS measurements   

To probe the dynamics of silica particles suspended in pure water, we collected 500 frames using the Pilatus detector operating with a 400 Hz frame rate (0.0002 s exposure and 0.0023 s read-out time per image frame).

In addition, the colloidal suspension in the water/glycerol solution was measured with 240 frames at 5 Hz using the Pilatus and FReLoN detectors, as well as 1024 frames at 200 Hz using only the Pilatus detector. Exposure times for a single frame were 0.002 s (200 Hz) or 0.02 s (5 Hz), respectively.

In the case of the FReLoN detector, the measurements were dark image subtracted before further evaluation. As the Pilatus detector has no dark current, this procedure was not required. Both detector images were flatfield corrected and unusable pixels were masked. Data processing was carried out using the *PYXPCS* software package developed at ID10, ESRF. The *q* values for the calculation of autocorrelation functions, 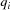, and the spacing between them, 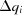, were optimized by varying these input values, and the smallest values which still gave reasonable statistics were chosen.

## 4. Results and discussion   

### 4.1. XPCS in pinhole–USXAS geometry   

**Figure 2:**
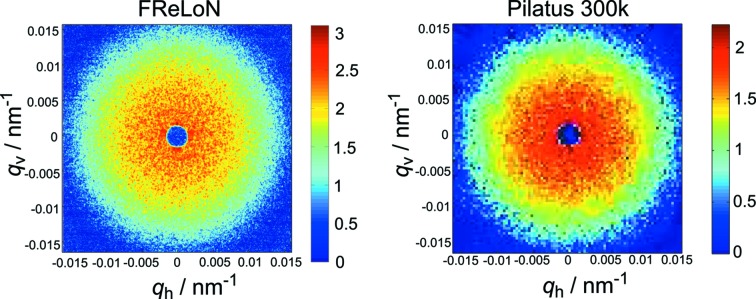
Speckle pattern of the same sample of 450 nm silica particles in water measured with the FReLoN and Pilatus detectors. The exposure time for both images was 0.2 ms. The beamstop has a diameter of 1.6 mm.

The utilization of a pinhole scattering geometry allows the use of the full, multi-speckle pattern of the scattered intensity on a large range of scattering vectors. A typical speckle pattern from a sample of 450 nm silica particles in water is shown in Fig. 2 ▸, measured with both available detectors and an exposure time of 0.2 ms each. Due to the higher resolution, the speckle pattern in the scattered intensity is much more clearly visible in the image using the FReLoN detector.

**Figure 3:**
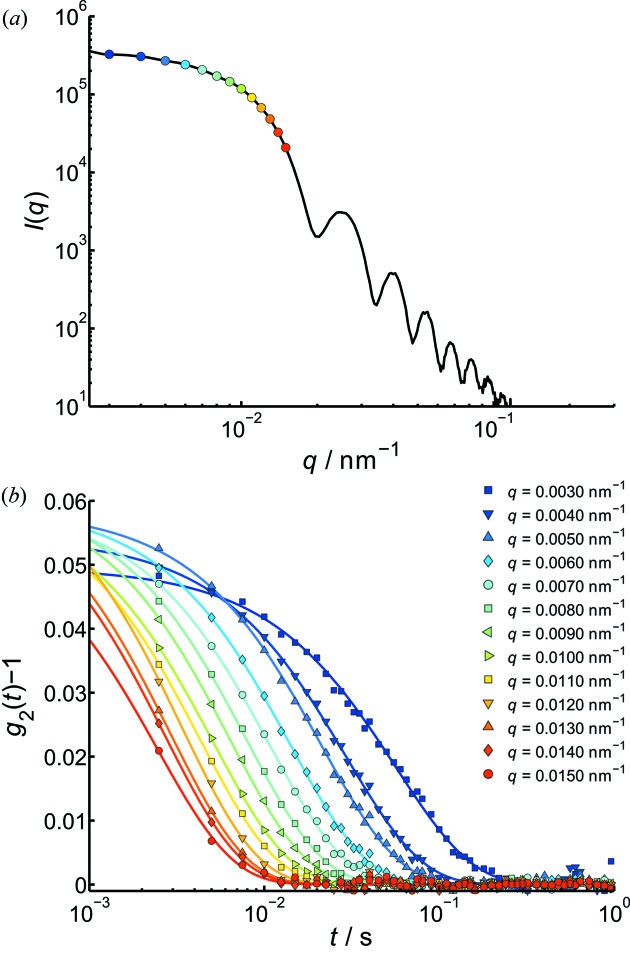
(*a*) Static scattering intensity and (*b*) intensity autocorrelation functions of a sample of 450 nm-diameter silica particles in water measured with the Pilatus 300K detector. A contrast 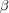 = 0.05–0.06 was measured. Additional (coloured) data points in (*a*) correspond to the *q*-values of the 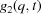 functions in (*b*).

Due to the fast dynamics of colloids in pure water, XPCS measurements on this sample can only be obtained using the Pilatus detector. Fig. 3(*a*) ▸ shows the corresponding small-angle scattering profile of the 450 nm silica particles in water, which was calculated by azimuthally averaging all measured frames. The corresponding autocorrelation functions are displayed in Fig. 3(*b*) ▸. For this sample, the smallest *q* value at which an autocorrelation function can still be calculated was found to be about 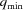 = 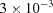
 nm^−1^ with a range of 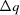 = 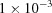
 nm^−1^. Even though the static scattering curve can be measured at smaller values, the corresponding correlation functions could not yield a proper baseline. Note that the determination of the lowest possible *q* value is not given by a strict criterion, as many different parameters such as the scattering power of the sample in addition to the angular resolution of the detector and size of the beamstop. In general it can be stated that, due to the small number of pixels near to the beamstop, a sufficiently scattering sample is needed in order to calculate successfully the correlation functions at this small *q* value.

To analyze lower scattering samples at smaller *q* values, a higher experimental resolution with a larger number of pixels in this area of the detector is needed. This will be further discussed in §4.2.

The highest *q* is only given by the maximum acquisition rate of the detector, as the obtained correlation functions are still of good statistics but, as the correlation rate increases with 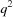, they become too fast to be measured.

**Figure 4:**
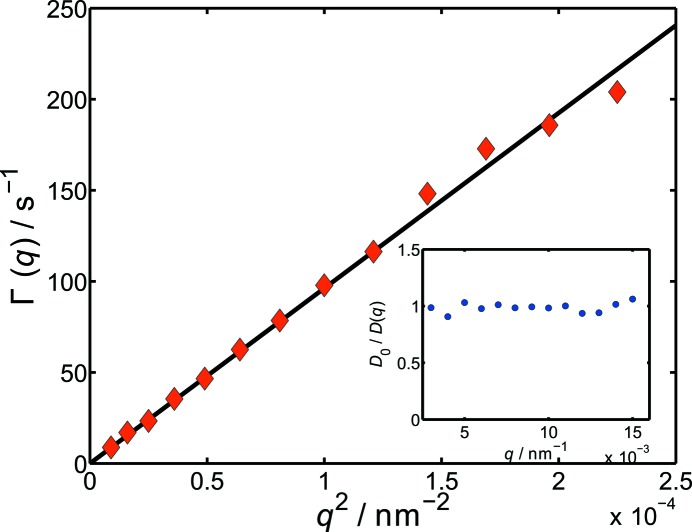
Results of the fits with equation (7) to the data in Fig. 3 ▸. A linear relation between Γ and 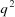 can be seen, corresponding to purely diffusive particle dynamics. Inset: 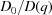 plotted *versus* 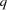. 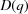 is the measured diffusion coefficient at each *q*, with 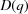 = 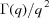.

The plotted autocorrelation functions were fitted using a single exponential function [equation (7)], which presents the measured data very well (solid lines). The obtained contrast was found to be around 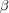 = 0.05–0.06. In general, a reduction of the contrast was found for the smallest accessible *q*-values (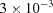 to 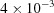
 nm^−1^), which is caused primarily by the reduced number of pixels and residual parasitic background. The possible heterodyne effect due to the higher background scattering near the beamstop is not severe since the obtained 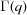 values are consistent with the expected homodyne decay rates. These decay rates 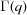, given by the exponential fits, are plotted against 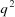 in Fig. 4 ▸.

Clearly, a linear relation between Γ and 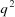 can be obtained, which arises from the purely diffusive behaviour of the particles dynamics and therefore demonstrates the good performance of the presented setup. Calculating the hydrodynamic radius from the linear refinement using equation (9) gives 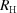 = 239 nm, which is in reasonable agreement with the value from the static scattering (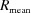 = 225 nm).

**Figure 5:**
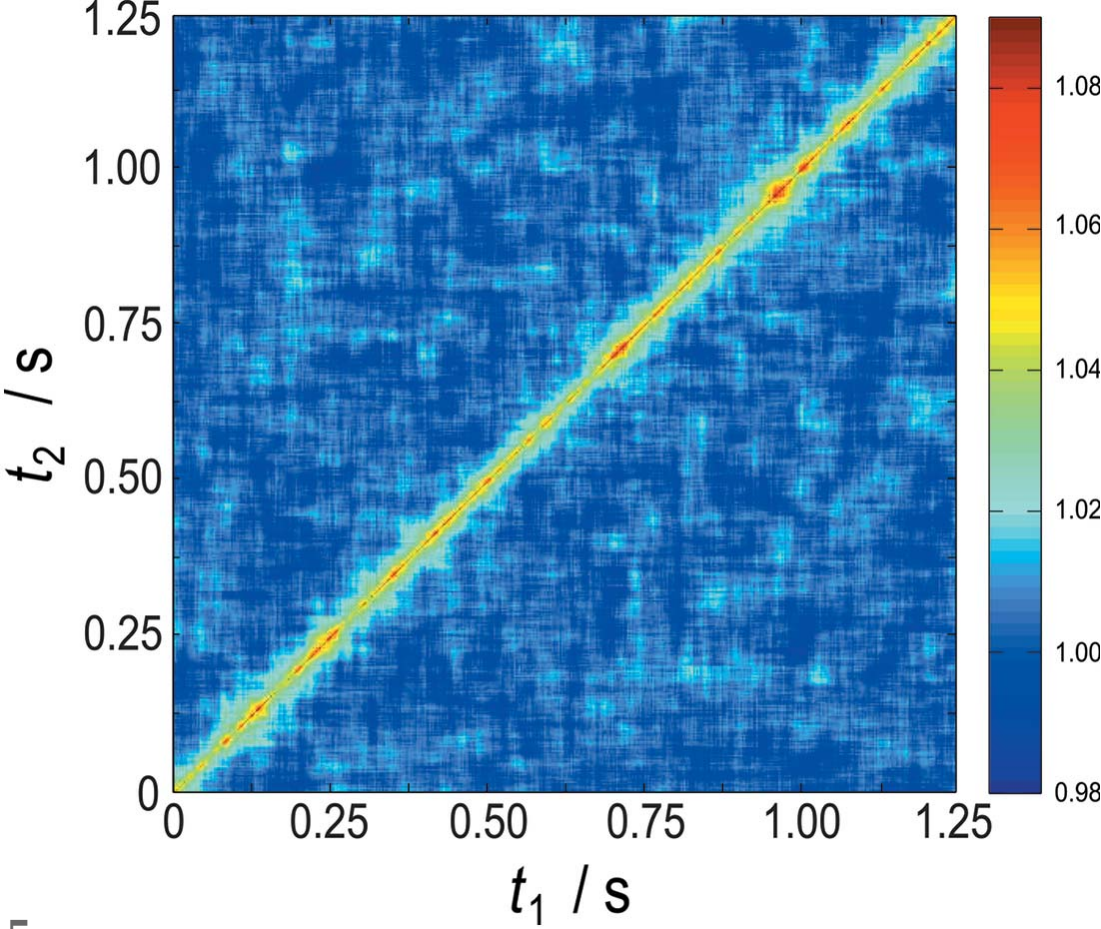
Two-time correlation function 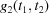 at 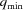 = 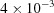
 nm^−1^.

A certain advantage of a two-dimensional detection scheme is the fast acquisition of a large amount of data due to the large number of pixels. This increases not only the statistical quality of the data and considerably decreases the measurement time but allows also a better ensemble averaging of the correlation functions. Furthermore, additional quantities such as the calculation of a two-time correlation function from the measured data can be performed. The two-time correlation function 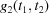 of the previous displayed data is calculated for a scattering vector 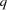 = 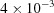
 nm^−1^ and shown in Fig. 5 ▸. As expected for a purely diffusive system, no time evolution (aging) can be seen in these data. Furthermore, it shows that no radiation damage is present or the sample is not evolving during the measurement.

### 4.2. Comparison between different detection schemes   

**Figure 6:**
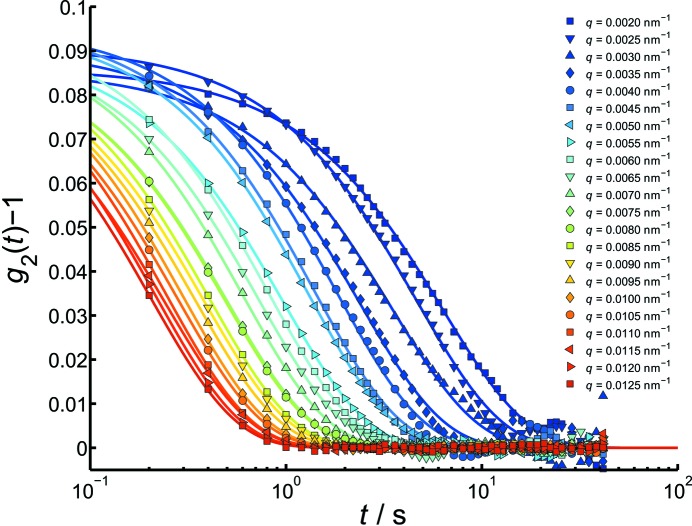
Intensity autocorrelation function of 600 nm silica particles in water/glycerol measured with the FReLoN detector at 5 Hz.

In order to investigate the influence of the detector characteristics on the quality of the obtained XPCS data, measurements were additionally performed using the Pilatus and the FReLoN detectors. The silica particles were suspended in a glycerol/water solution to slow down the dynamics of the colloidal system. The obtained correlation functions are displayed in Figs. 6 ▸ and 7 ▸.

The two detectors differ in two principle characteristics. The Pilatus is a fast detector (400 Hz), allowing access to smaller correlation times *t*, but has rather large pixels. The FReLoN is slower (5 Hz), but it has seven times smaller pixels. This smaller pixel size of the FReLoN detector has two impacts on the obtained data. In general, a smaller pixel size can be translated as a higher angular resolution of the experiment. In the case of the Pilatus 300K, each speckle is within one pixel, while the same speckle distributes over a cluster of pixels on the FReLoN detector. This should result in a higher contrast β. That this is the case can directly be demonstrated with the contrast values obtained from the 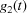 data. The contrast obtained by using the Pilatus detector is 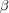 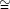 0.04, whereas the higher angular resolution of the FReLoN detector gives a contrast of 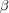 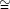 0.08–0.09.

Furthermore, a much smaller 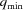 can be chosen in the data treatment process as more pixels near to the beamstop contribute to the signal (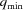 = 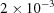
 nm^−1^ for the FReLoN and 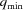 = 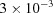
 nm^−1^ for the Pilatus detector). As the correlation rate is proportional to 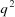, these correlation functions exhibit also much slower decay rates. Furthermore, due to the smaller pixel size and therefore larger number of pixels, a smaller sectioning (FReLoN: 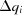 = 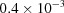
 nm^−1^; Pilatus: 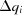 = 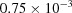
 nm^−1^) can be chosen.

**Figure 8:**
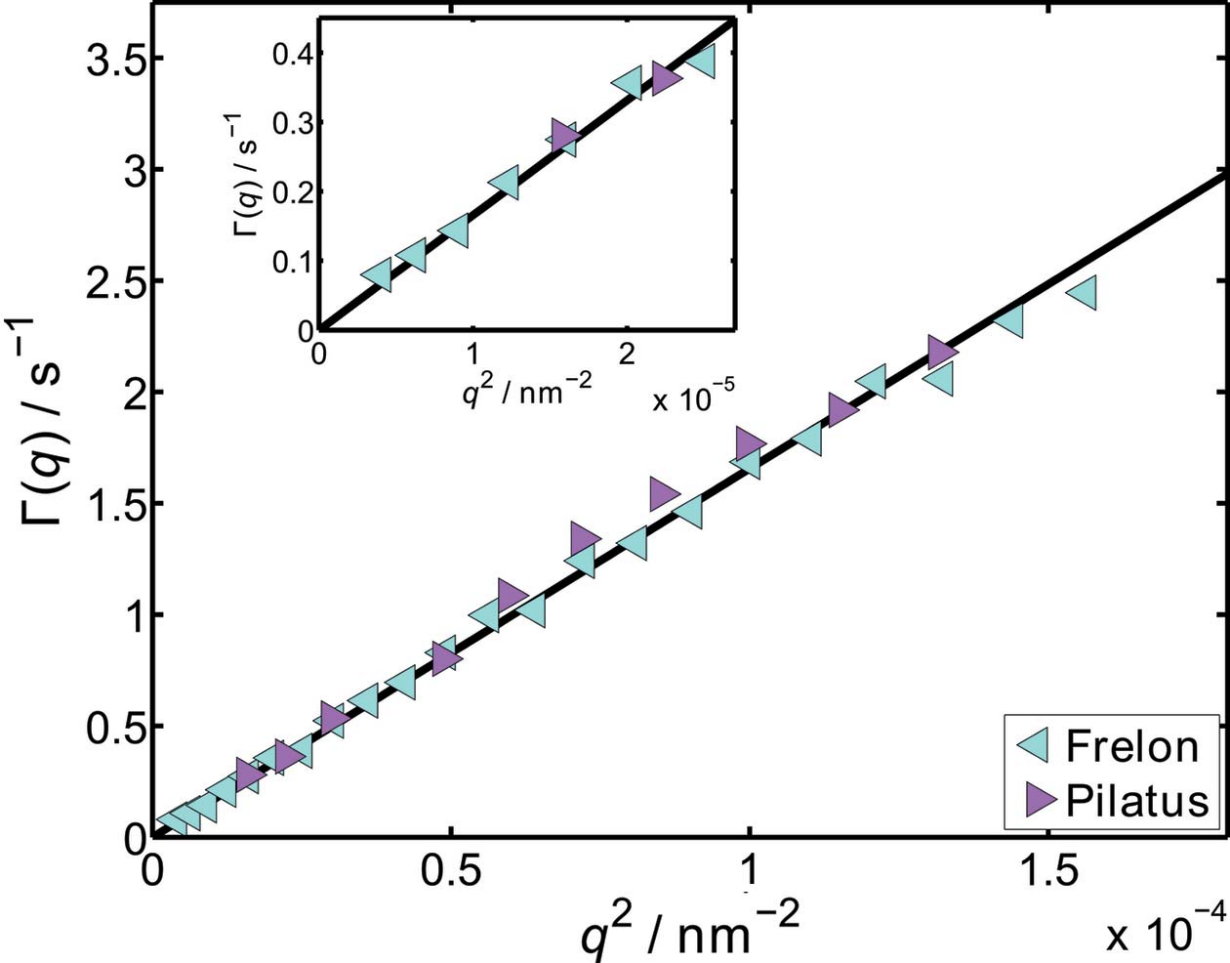
Decay rates of the 600 nm silica particles in a glycerol/water solution obtained with the two different detectors. The inset shows a zoom into the low *q* region.

The resulting correlation rates are displayed in Fig. 8 ▸. The data points are fitted and the hydrodynamic radius obtained was found to be 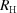 = 316 nm. No quantitative difference between the data obtained with the two different detectors can be observed. It clearly shows, however, the advantage of a higher angular resolution of the experimental setup, as the correlation rates can be determined on a larger *q* range and with a smaller step size. Especially when accessing the hydrodynamic structure factor from the XPCS data, this feature may be an advantage. It can be stated that a higher angular resolution, even though with limited temporal resolution, might give data of higher quality.

**Figure 9:**
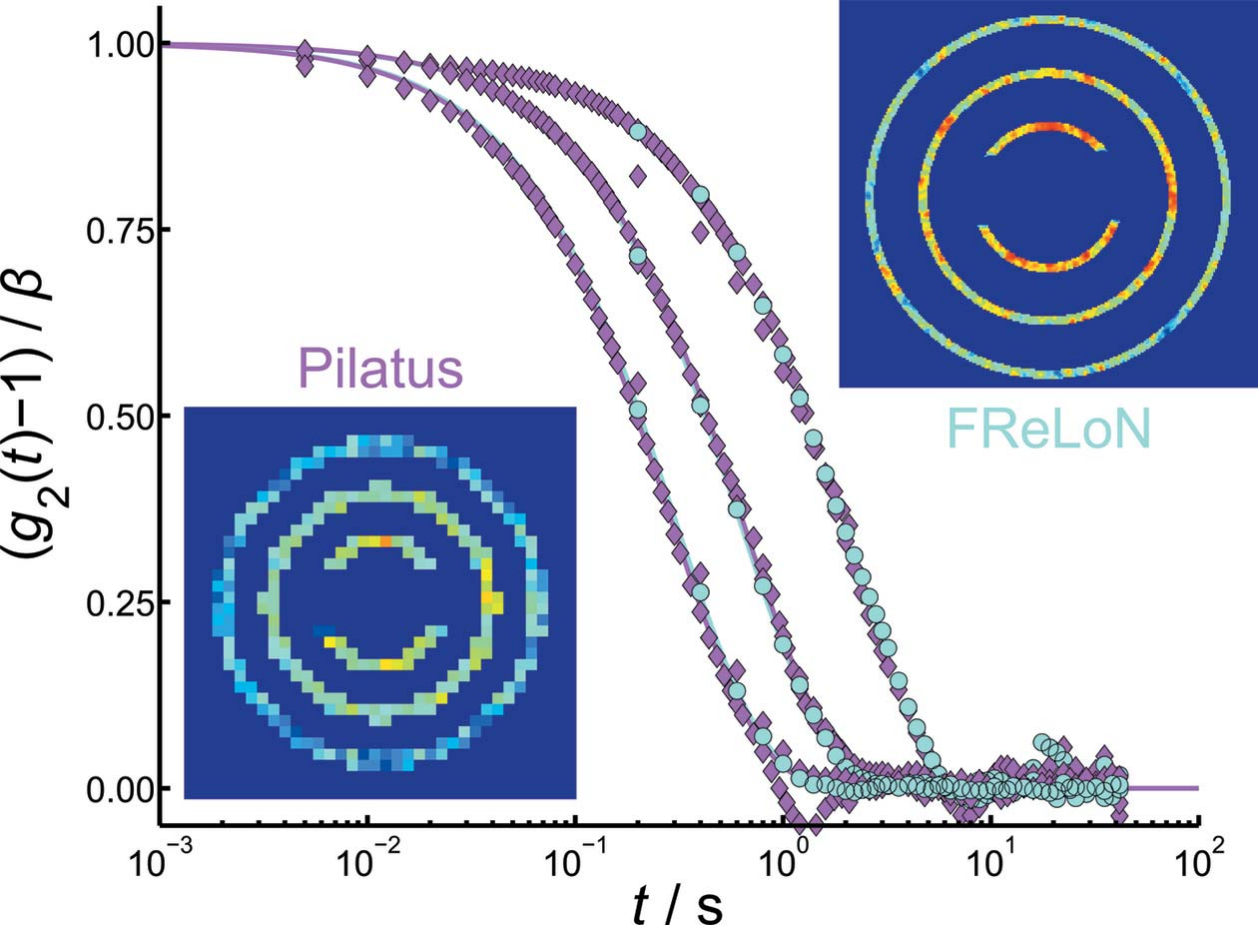
Intensity autocorrelation function obtained with the Pilatus 300K and FReLoN detector at 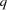 = 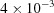
 nm^−1^, 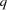 = 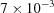
 nm^−1^ and 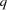 = 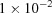
 nm^−1^. The insets show the corresponding two-dimensional scattering patterns.

In the following, the data given by the two detectors are further compared. Fig. 9 ▸ shows three representative correlation functions at 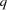 = 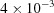
 nm^−1^, 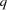 = 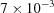
 nm^−1^ and 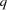 = 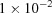
 nm^−1^ each measured with the two detectors. The insets show the corresponding detector image, where only the pixels corresponding to the three *q* ranges are not masked. The higher number of pixels corresponding to the signal of each *q* range in the case of the FReLoN detector is obvious. The sample-to-detector distance is the same for both detectors.

According to equation (4), the probability distribution of intensity 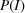 in one of the displayed rings should follow an exponential decay in the case of a fully coherent experiment, *i.e.* 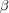 = 1. For experiments with lower coherence (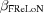 ≃ 0.09 and 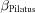 ≃ 0.04 as obtained from the correlation functions), the distribution of intensities should follow equation (5).

**Figure 10:**
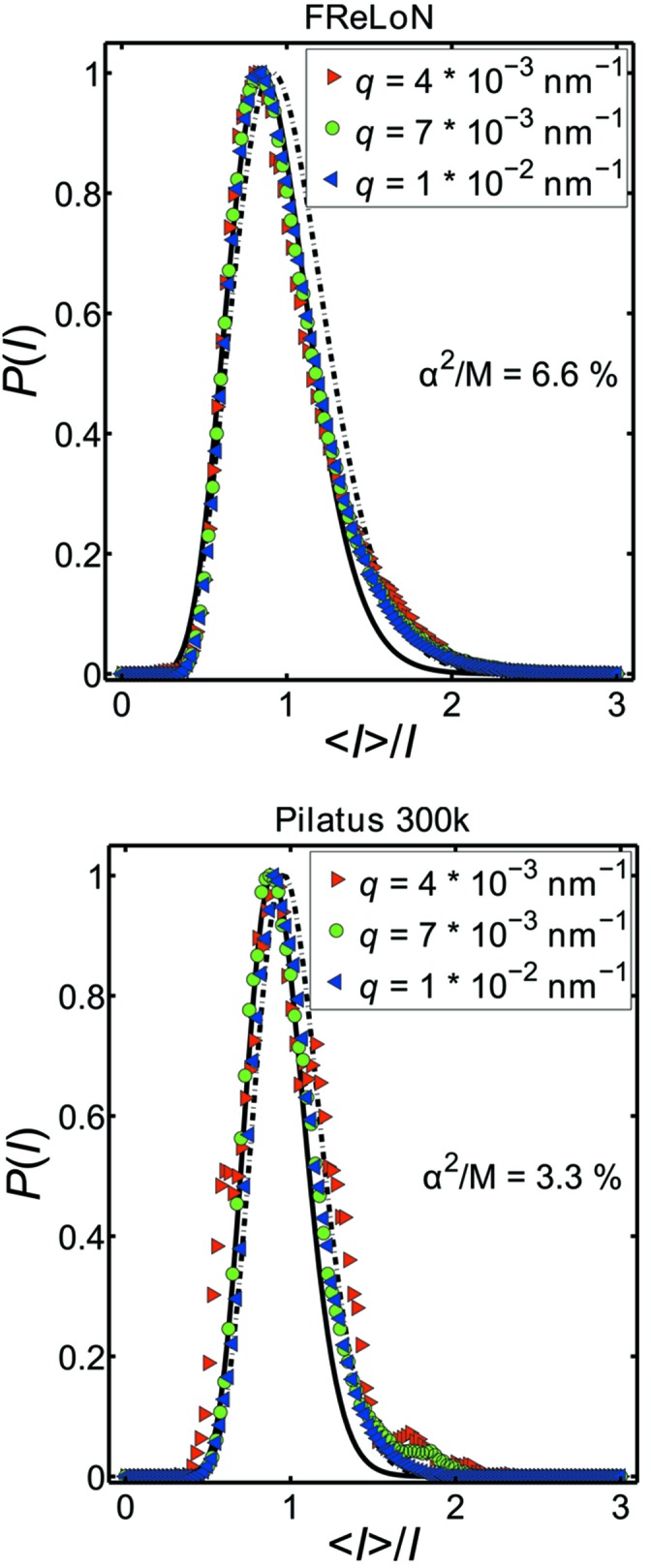
Intensity distributions 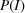 at 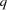 = 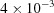
 nm^−1^, 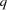 = 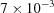
 nm^−1^ and 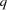 = 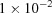
 nm^−1^ measured with the FReLoN and Pilatus detectors, respectively.

In Fig. 10 ▸, the intensity distributions measured with the two detectors are shown for 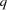 = 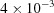
 nm^−1^, 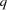 = 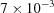
 nm^−1^ and 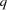 = 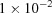
 nm^−1^. The distributions were calculated from each two-dimensional image separately and then the distributions from all frames were averaged to achieve better statistics [FReLoN: 240 frames; Pilatus: 240 frames (5 Hz) + 1024 frames (200 Hz)]. The resulting distributions were fitted using equation (5), with 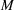 = 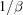 being the only free parameter (dashed line). Only a moderate agreement with the data can be obtained. The agreement can be improved, however, if a constant value 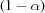 is subtracted from the scaled intensity before the mean value is calculated (continuous line). This procedure corresponds to the assumption that a constant part of the incident beam does not contribute to the coherent interference which results in the speckle pattern (Abernathy *et al.*, 1998 ▸; Grübel *et al.*, 2008 ▸). In our case, we found that a value of 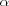 = 0.925 is needed to sufficiently describe the data. The contrast is calculated as 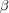 = 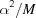 (Grübel *et al.*, 2008 ▸), resulting in 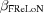 = 0.066 and 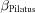 = 0.033. In both cases, slightly smaller values than the previously obtained contrasts from refining the correlation functions are found. However, the roughly doubled contrast of the FReLoN detector is evident. Furthermore, the intensity distributions are not significantly modified and follow the prediction even though fundamentally different detection principles are involved. It can be stated that an integrating CCD-type detector having single-photon sensitivity can be used for XPCS experiments and both detectors show similar statistical properties. Due to the reduced pixel size and therefore higher angular resolution, the FReLoN detector even provides better results for samples with slower dynamics and non-equilibrium systems.

## 5. Concluding remarks   

We have demonstrated the feasibility of performing high-quality XPCS measurements in the USAXS range using a high-resolution pinhole collimation instrument with 1 Å X-ray wavelength. The correlation functions can be obtained with high resolution in much shorter measurement times as compared with previous setups using a Bonse–Hart USAXS camera. The presented setup offers several new possibilities, like studying non-equilibrium systems and obtaining two-time correlation functions over the scattering vector range comparable with small-angle light scattering. We would like to point out that several experimental applications may become accessible with this new setup, which were out of the feasible range of DLS and XPCS. Examples include the dynamics of self-propelled colloids, local velocity fluctuations in concentrated sedimenting suspensions, collective movement in ageing colloidal gels, dynamics of drying colloidal film such as paint, and so on.

Furthermore, it has been demonstrated that two detectors with different X-ray detection principles can successfully be used to measure XPCS data. The high time resolution of the Pilatus and the high angular resolution of the FReLoN detector are complementary for different kinds of samples. The obtained data were found to be of similar statistical quality. A further improvement in the future may be achieved with more advanced detectors, combining high time and high spatial resolution, like for example with the Eiger pixel detector.

## Figures and Tables

**Figure 7:**
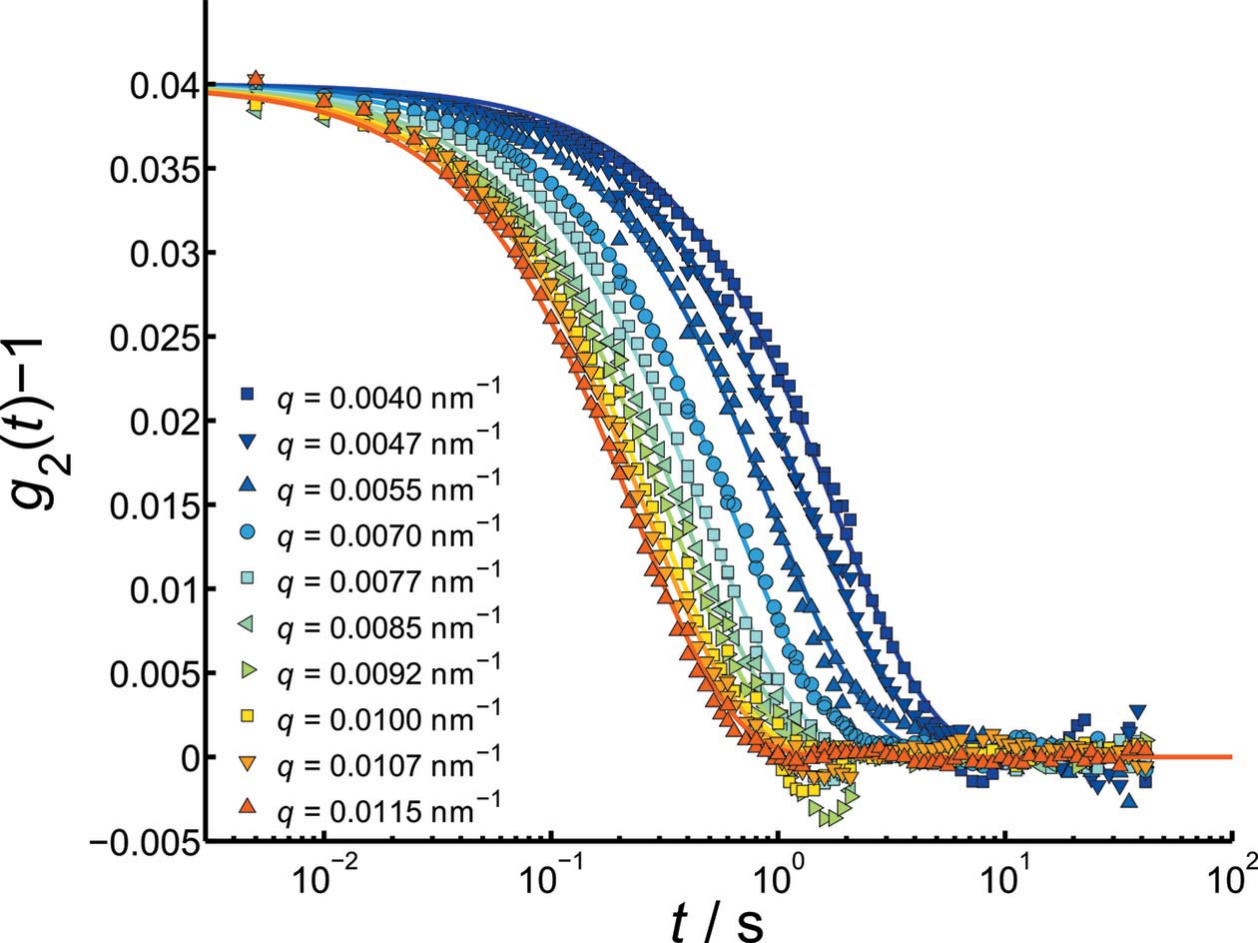
Intensity autocorrelation function of 600 nm silica particles in water/glycerol measured with the Pilatus detector at 200 Hz and 5 Hz.
